# Porphyrins are increased in the faeces of patients with prostate cancer: a case-control study

**DOI:** 10.1186/s12885-018-5030-1

**Published:** 2018-11-12

**Authors:** Daniel Riani Gotardelo, Lilia Coronato Courrol, Maria Helena Bellini, Flávia Rodrigues de Oliveira Silva, Carlos Roberto Jorge Soares

**Affiliations:** 1 Instituto de Pesquisas Energéticas e Nucleares, IPEN-CNEN/SP, Av. Prof. Lineu Prestes, 2242- Cidade Universitária, São Paulo, 05508-000 Brazil; 2 Instituto Tocantinense Presidente Antônio Carlos - ITPAC Palmas, Tocantins, Brazil; 3 Instituto de Ciências Ambientais Químicas e Farmacêuticas (ICAQF), Departamento de Física (DF), UNIFESP, Campus Diadema, São Paulo, Brazil

**Keywords:** Porphyrin, Biomarker, Faeces, Prostate Cancer, Fluorescence spectroscopy

## Abstract

**Background:**

Experimental models of prostate cancer have demonstrated increased levels of protoporphyrin IX (PpIX) in the blood and faeces of mice. Hence, the quantification of these autofluorescent molecules could be hypothesized to be a potential marker for this type of tumour. In this case-control study, the autofluorescence of porphyrins in human faeces from patients with prostate cancer and control subjects was analysed using fluorescence spectroscopy.

**Methods:**

First, 3 mL of analytical-grade acetone was added to 0.3 g of faeces, and the mixture was macerated and centrifuged at 4000 rpm for 15 min. The supernatant was analysed spectroscopically. The emission spectra from 550 to 750 nm were obtained by exciting the samples at 405 nm.

**Results:**

A significant difference between the samples from control and cancer subjects was established in the spectral region of 670–675 nm (*p* = 0.000127), which corresponds to a significant increase in faecal porphyrins in patients with cancer. There was no statistically significant correlation between PSA levels and faecal porphyrins.

**Conclusion:**

In this preliminary study conducted in humans, the results show a simple and non-invasive method to assess faecal porphyrins, which have the potential to function as a tumour biomarker in patients with prostate cancer. This approach has improved sensitivity and specificity over PSA testing. Additional prospective studies with larger sample sizes are required to validate these findings.

**Electronic supplementary material:**

The online version of this article (10.1186/s12885-018-5030-1) contains supplementary material, which is available to authorized users.

## Background

Other than skin cancer, prostate cancer is the most common type of cancer among men. In 2013, there were 1.4 million cases of prostate cancer and 293,000 deaths. The probability of developing prostate cancer is 1 in 15 between birth and the age of 79 years [1]. Commonly used methods to screen for this disease are the prostate-specific antigen (PSA) measurement and the digital rectal examination.

PSA is used as a marker for screening, early diagnosis, treatment response assessment, and tumour progression determination. PSA is also prostate-specific but not disease-specific. PSA dosage has several limitations, such as (1) low specificity for the detection of early-stage tumours; (2) the possibility of altered results related to medications, inflammation (benign prostatic hyperplasia and prostatitis), age, trauma and urologic manipulation; and (3) controversy related to the marker level that indicates the need for a biopsy or reveals the presence of metastasis. The discovery of new biomarkers that may or may not be associated with PSA using molecular biology techniques could potentially revolutionize the diagnosis and management of prostate cancer [2].

Clinical and experimental studies have been conducted to quantify substances that could be capable of identifying the presence of several types of tumours, including prostate tumours. Some of these substances, called endogenous fluorophores, are autofluorescent and are identifiable through fluorescence spectroscopy studies. Several fluorophores have been quantified in cells and tissues, but there are a limited number of papers addressing the possibility of measuring these components in human biological fluids for cancer diagnosis. The known fluorophores include collagen, elastin, tryptophan, tyrosine, phenylalanine, pyridoxine, and porphyrin derivatives [3].

**Fig. 1:**
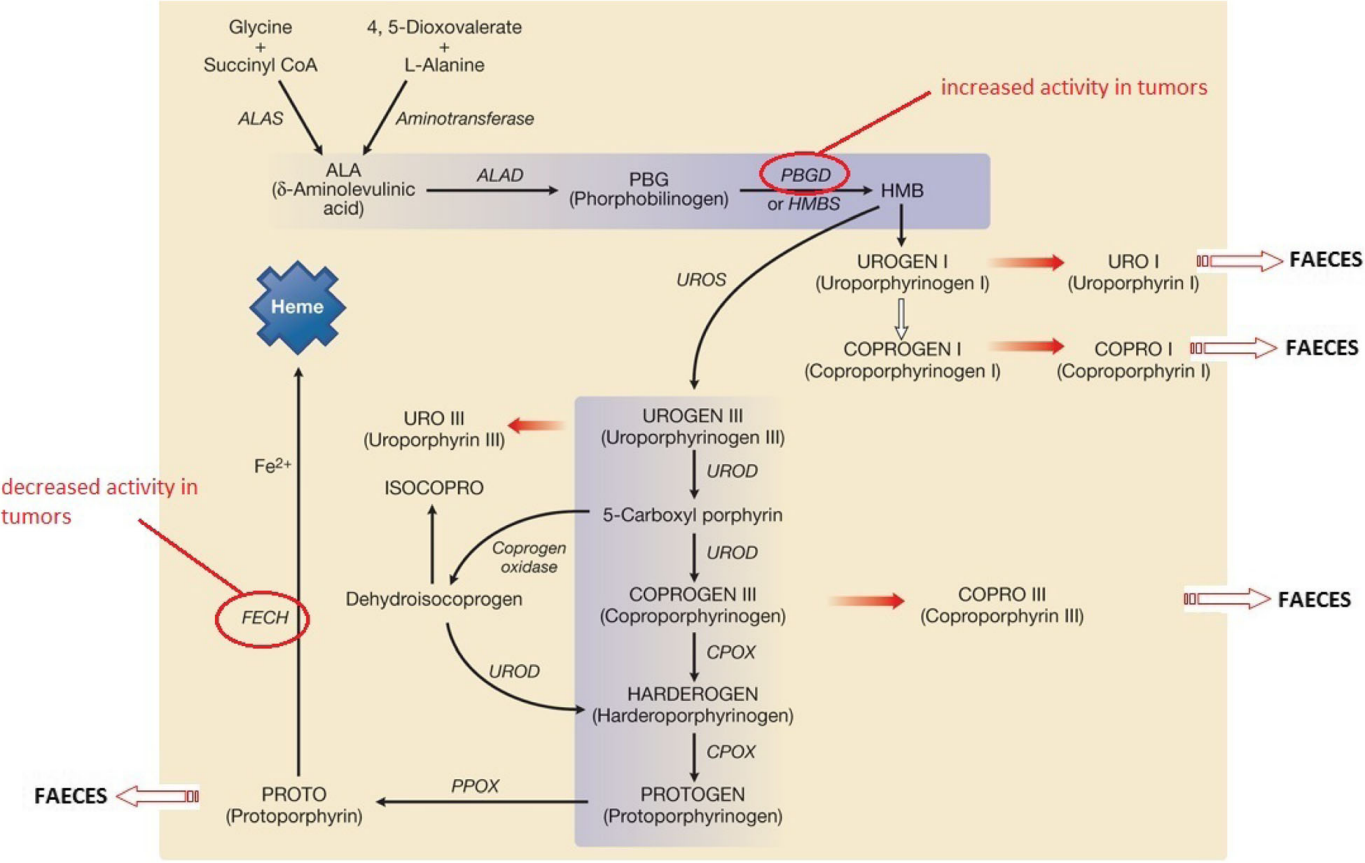
Biosynthesis of haeme and enzymes possibly altered in tumors [23]. *ALAS =* ALA synthase; *ALAD =* ALA dehydratase (Porphobilinogen synthase); *PBGD =* Porphobilinogen deaminase; *HMB =* Hydroxymethylbilane; *UROS =* Uroporphyrinogen synthase. *UROD =* Uroporphyrinogen descarboxilase; *CPOX =* Coproporphyrinogen oxidase; *PPOX =* Protoporphyrinogen oxidase; *FECH =* Ferrochelatase

Porphyrins are cyclic tetrapyrrolic compounds that act as intermediates in haem biosynthesis (Fig. 1). The most important compounds in nature are coproporphyrin, uroporphyrin, and protoporphyrin IX (PpIX), which are produced during haem synthesis. Abnormal metabolism of the erythrocyte PpIX has been observed in individuals with cancer who show elevated levels of this substance. It may be quantified by fluorescence in the tissues and biological fluids of human and guinea pigs that have been inoculated with neoplastic cells. The determination of the levels of this substance has been used to assess breast and bowel cancer in humans and prostate cancer in animal models [3, 4–9].

There are several main hypotheses that seem to explain the alteration in porphyrin metabolism in cancer. One is the hypervascularization typical of malignant neoplasia, which could potentially result in the accumulation of porphyrins due to a greater amount of haemoglobin reacting with the tumour in an attempt to neutralize it [5]. PpIX also has higher fat solubility in an acidic environment, which is compatible with the presence of lactic acid from anaerobic metabolism found in several tumours [4]. The presence of bacteria in hollow viscera tumours could also degrade haemoglobin on the luminal surface, which increases the porphyrin autofluorescence signal [10]. Most important of these hypotheses are the metabolic abnormalities present in malignant cells. Intracellular iron availability is reduced due to the increasing frequency of cell division, decrease in the activity of the ferrochelatase enzyme, and increase in the activity of the porphobilinogendeaminase enzyme. This process interferes with iron incorporation during haeme biosynthesis and favours the accumulation of PpIX in the tumour (Fig. 1) [7, 10].

A significant difference could be established between PpIX autofluorescence in healthy and prostate cancer animal models. In animals with tumours, PpIX synthesis is altered, which probably interferes with the synthesis of coproporphyrin and other faecal metabolites [5, 6]. In this study, we analysed faecal porphyrin levels in subjects with prostate cancer in a case-control study with humans.

## Methods

### Study type and sampling

In this case-control study, we recruited individuals with and without prostate cancer. All prostate cancer patients had a diagnosis confirmed by biopsy at Hospital Márcio Cunha/Fundação São Francisco Xavier’s Oncology Division, Ipatinga, MG, Brazil, and we collected their material before the start of treatment. For the controls, we selected healthy individuals who were subjected to digital rectal examination and PSA measurement with negative results in the last 12 months. They were recruited within the Municipal Health Secretary Urology Ambulatories in the city of Ipatinga, MG, Brazil. Recruitment was carried out to obtain proper matching between attributes such as age, ethnicity, degree of schooling, socioeconomic status, and other variables.

### Clinical and sociodemographic variables

In addition to porphyrias, there is a group of metabolic diseases in which there could be an increase in faecal porphyrins. The investigation for other conditions that could cause this increase was conducted through clinical interviews and evaluation of patients’ laboratory tests. Sociodemographic variables were analysed to guarantee homogeneity among the studied groups. All patients filled out a research form and consented to providing their faeces for the study. Table 1 shows the groups’ homogeneity from the analysis of the studied variables.

**Table 1:** Studied variables frequency in cases and controls

Variables	Controls		Cases	
	*n*	%	*n*	%
Marital Status				
Married	6	66.6	7	77.7
Single	1	11.1	0	0
Divorced	0	0	0	0
Widowed	2	22.3	2	22.3
Alcoholism				
Yes	1	11.2	0	0
No	8	88.8	9	100
Smoking				
Yes	0	0	3	33.3
No	9	100	6	66.6
Family history of prostate cancer				
Yes	2	22.3	2	22.3
No	7	77.7	7	77.7
Personal history of other kinds of cancer				
Yes	0	0	0	0
No	9	100	9	100
Prostatic disease				
Yes	0	0	0	0
No	9	100	9	100
Diabetes				
Yes	2	22.3	1	11.2
No	7	77.7	8	88.8
Dyslipidemia				
Yes	1	11.2	1	11.2
No	8	88.8	8	88.8
Porphyria				
Yes	0	0	0	0
No	9	100	9	100
Anemia				
Yes	0	0	0	0
No	9	100	9	100
Lead/metals intoxication				
Yes	0	0	0	0
No	9	100	9	100
Antibiotics use				
Yes	0	0	0	0
No	9	100	9	100

The mean age ± SD in the prostate cancer patient group was 70.6 ± 7.75 years and in the control group was 66 ± 7.18 years (*p* value = 0.2).

### Porphyrin extraction

Faecal samples were collected in proper amber flask receptacles without exposure to light, stored, and transported at − 20 °C prior to analysis. According to a thermometer installed inside the Styrofoam boxes, the cold temperature was maintained. All the materials were analysed within 48 h of collection.

A scan was carried out on four dilutions to choose the best volumes of acetone and faeces to obtain the most intense fluorescence. The assayed concentrations are shown in Table 2. For this optimization, two patients from each group were studied, and samples were prepared in triplicate.

**Table 2:** Amount of faeces and acetone used in each dilution

Dilution	Acetone (mL)	Feces (g)
1	3	0,3
2	1	0,05
3	3	0,2
4	3	0,4

### Fluorescent spectral analysis

The emission spectra were obtained by exciting the samples at 405 nm inside a 1-mm optical path cuvette. The samples’ fluorescence was analysed using a Horiba Jobin Yvon Fluorolog 3 Fluorimeter in the range of 550–750 nm. The material was analysed in triplicate, and the mean of the three measures of each sample was considered for calculating the results (average multiple curves).

Data were analysed using descriptive and inferential statistics. For spectra analysis, we used the software OriginPro 8® version 8.0724. For statistical analysis, we used the software SPSS® version 19.0. Quantitative variables are presented as means and standard deviations. Qualitative variables are presented as absolute and relative frequencies. A Kolmogorov-Smirnov test was applied for normality verification. We used an independent t-test for the comparison of group means and a Pearson test to verify correlations. A significance level of 5% was adopted.

## Results

Because this study is the first to quantify porphyrin in the faeces of humans with prostate cancer, a preliminary study was carried out with 9 individuals in each group, which is a similar sample size to those used in other studies [6, 11, 12]. Table 3 shows the result of the sample calculation based on the mean and standard deviation of two studies that have already demonstrated a difference between the porphyrin levels of cases and controls. Because this significant difference, a very small sample was estimated necessary for this study.

**Table 3:** Reference studies containing mean concentration ± SD of porphyrins found in cases and controls

Reference study	Study type	Control group (mean ± SD)	Case group (mean ± SD)	Confidence Level	Power	Calculated sample
Study of Protoporphyrin IX Elimination by Body Excreta: A new Noninvasive Cancer Diagnostic Method. [6]	Experimental (Prostate cancer)	0.72 ± 0.12	2.73 ± 0.51	99%	90%	4
Concentration of protoporphyrin IX in cancer tissues and blood in patients with colorectal cancer at early stage. [4]	Clinical (Colorectal cancer)	0.58 ± 0.08	2.28 ± 0.10	99%	90%	2

**Fig. 2:**
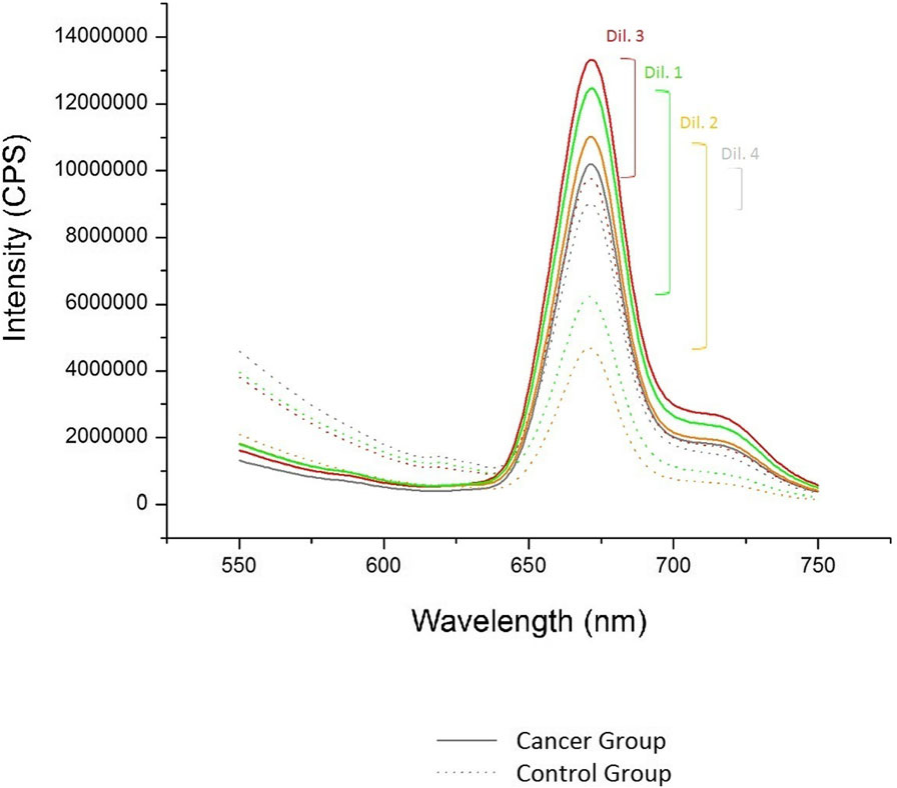
Difference between spectrophotometric peaks obtained from each dilution

An initial study was carried out to determine the best acetone volume and faeces mass to use for optimal porphyrin fluorescence intensity in samples from the cancer patient and control groups (Additional file 1: spreadsheet 1). The results are shown in Fig. 2.

Dilutions 1 and 2 showed greater differences between groups and dilution 1 was chosen for later analyses.

**Fig. 3:**
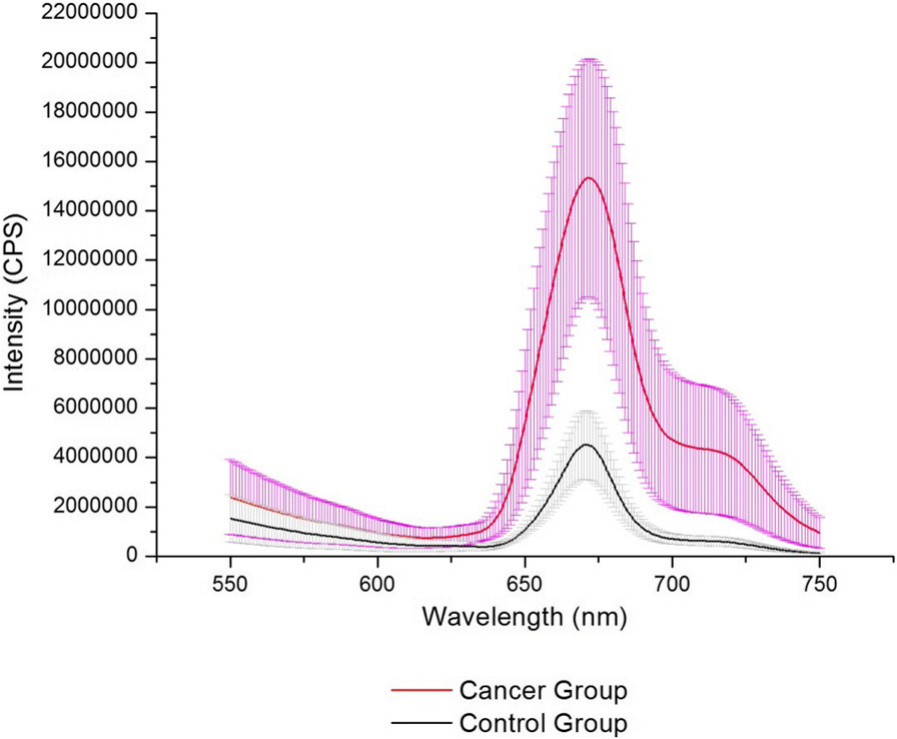
Analysis of porphyrins extracted from the feces of control and cancer groups

Figure 3 shows the average results of the porphyrin fluorescence analysis of the control and cancer patient groups (Additional file 1: spreadsheets 2 and 3). The fluorescence intensity of faecal porphyrins was significantly higher in the group of patients with cancer. The Kolmogorov-Smirnov test indicated a normal distribution. For mean comparisons, we used an independent t-test with a significance level of *p* < 0.05.

The difference in mean maximum emission intensities at 671 nm between the control and cancer patient groups was significant (Fig. 3), at 4.13E + 06 CPS (±1.29E + 06 CPS) for controls and 1.50E + 07 CPS (±4.94E + 06 CPS) for cancer patients, with a *p* value of 0.000127.

For further study of cancer patients, we recruited 3 patients from each D’Amico classification stage, with PSAs ranging from 4.41 ng/mL to 71.05 ng/mL and Gleason scores ranging from 4 to 9 within each stage. However, there was no correlation between any of these variables and the levels of porphyrins in the faeces of these cancer patients. A potential correlation between the PSA levels and faecal porphyrins was assessed using Pearson’s coefficient (r). There was no statistically significant correlation between these variables (*R* = − 0.0221).

## Discussion

Currently, the main challenges in prostate cancer are finding alternatives to PSA measurement for diagnosis and researching new markers that can predict the disease’s aggressiveness. This would assistin therapeutic planning and avoid unnecessary interventions, for example, the need for prostate biopsy and its associated risks for diagnosis. Determining the accuracy of PSA testing has been difficult because most men with normal PSA values will not undergo biopsy unless their digital rectal exam is abnormal. This workup bias tends to overestimate sensitivity and underestimate specificity of PSA testing. Performance can also be overestimated because PSA often detects clinically unimportant cancers. Additionally, protocols that use large numbers of biopsies to evaluate patients with elevated PSA levels may be detecting incidental cancers that were not the aetiology of the PSA elevation. In this context, the search for new biomarkers capable of improving the sensitivity and specificity of PSA testing is necessary [13–15].

There are several advantages to demonstrating that a biomarker for prostate cancer can be quantified from faeces, such as low cost, decrease in discomfort related to blood withdrawal, and negation of the need for specialized professionals and materials for the collection procedure as well as the clinical community’s familiarity with performing this kind of exam for other purposes (e.g., parasitological examination and faecal occult blood testing). On the other hand, many people might prefer to have a blood collection than to collect a faecal sample of themselves. Although many studies have shown an increase in blood porphyrins in other types of cancer, only a single experimental study in prostate cancer showed an alteration in faecal porphyrins. This study identified two emission bands in the faeces of nude mice [6]. One band at 632 nm had increased intensity and corresponded to the faecal concentration of PpIX. The other band at 671 nm corresponded to coproporphyrins and other faecal porphyrins, and the mice with prostate cancer had a lower intensity band compared to that found with the control group.

In the present study, only a single emission band at 670–675 nm was found in both the cancer patient and control groups. This band can be attributed to soluble porphyrins, such as coproporphyrin and uroporphyrin, or to the photoproducts formed by PpIX degradation. A peak corresponding to faecal PpIX was not found in our study. The main hypothesis to explain this could be the difference in the metabolism and concentration of different kinds of porphyrins in human faeces compared to those from nude mice. The same method of extraction and processing used in the nude mouse experiment was applied, but neither of this study’s groups presented a spectrophotometric peak at 632 nm. Another explanation for these findings could be the elapsed time gap from the collection of faeces to their analysis in the two studies. Despite being properly protected from light, the finding of a single spectrophotometric peak could be explained by the photodegradation of PpIX in our patient samples, as well as the increased activity of porphobilinogendeaminase enzyme in the cytoplasm of individuals with cancer. This enzymatic activity would have led to an increase in faecal coproporphyrins and uroporphyrins, as hypothesized by other authors [10].

An increase in faecal porphyrins has already been shown in other conditions, including porphyria (variegate porphyria and hereditary coproporphyria) and sideroblastic anaemia [16, 17]. The use of antibiotics is a common approach in clinical practice that could decrease the levels of faecal porphyrins by modifying intestinal flora [18]. The use of these medications excluded patients from our study. In this study, we were also careful to exclude conditions that could lead to false-positive results through clinical examination. The cholesterol and haemoglobin levels of all patients in both groups were verified [17, 19]. All patients were asked about their disease conditions, such as diabetes and dyslipidaemia, medication use (antibiotics), and the presence of any symptoms. This leads us to believe that the increase in faecal porphyrins indeed resulted from the altered metabolism of porphyrins caused by the tumour.

Predicting prostate cancer biological behaviour after being diagnosed is of great scientific relevance and importance for determining treatment. PSA is a tumour marker that is considered in risk predictor scores that are often used in clinical practice (Partin nomogram and D’Amico classification) [20, 21]. In the present study, we did not find a correlation between PSA level, Gleason score, D’Amico classification and the level of faecal porphyrins. However, there are in vitro studies correlating an increase in tumour size with an increase of PpIX blood levels in nude mice, which could ultimately lead to an increase in faecal porphyrins [5]. Nakayama et al. observed increases in PpIX in cancer cells, even during the latency stage that is characterized by no proliferation, no death, metabolic suppression, and the recovery of active status [22]. These findings suggest that faecal porphyrin concentrations could also be useful as biomarkers to monitor tumour growth and perhaps to assess the biological behaviour of this type of cancer.

## Conclusions

In conclusion, we have demonstrated in this preliminary study that faecal porphyrins have the potential to function as a tumour biomarker for prostate cancer. Additional prospective studies with larger sample sizes are required to validate these findings and elucidate the sensitivity and specificity of faecal porphyrins for the diagnosis of the disease.

## Additional file

Additional file 1: Spreadsheet 1- comparison between the dilutions used, spreadsheets 2 and 3 - results of the spectrophotometric peaks (XLSX 90 kb)
